# Sun Exposure Shapes Functional Grouping of Fungi in Cryptoendolithic Antarctic Communities

**DOI:** 10.3390/life8020019

**Published:** 2018-06-02

**Authors:** Claudia Coleine, Laura Zucconi, Silvano Onofri, Nuttapon Pombubpa, Jason E. Stajich, Laura Selbmann

**Affiliations:** 1 Department of Ecological and Biological Sciences, University of Tuscia, Viterbo 01100, Italy; coleine@unitus.it (C.C.); zucconi@unitus.it (L.Z.); onofri@unitus.it (S.O.); 2 Department of Microbiology and Plant Pathology, University of California, Riverside, CA 92521, USA; npomb001@ucr.edu; 3 Italian National Antarctic Museum (MNA), Mycological Section, Genoa 16166, Italy

**Keywords:** Antarctica, endolithic communities, fungal ecology, FUNGuild, ITS metabarcoding

## Abstract

Antarctic cryptoendolithic microbial communities dominate ice-free areas of continental Antarctica, among the harshest environments on Earth. The endolithic lifestyle is a remarkable adaptation to the exceptional environmental extremes of this area, which is considered the closest terrestrial example to conditions on Mars. Recent efforts have attempted to elucidate composition of these extremely adapted communities, but the functionality of these microbes have remained unexplored. We have tested for interactions between measured environmental characteristics, fungal community membership, and inferred functional classification of the fungi present and found altitude and sun exposure were primary factors. Sandstone rocks were collected in Victoria Land, Antarctica along an altitudinal gradient from 834 to 3100 m a.s.l.; differently sun-exposed rocks were selected to test the influence of this parameter on endolithic settlement. Metabarcoding targeting the fungal internal transcribed spacer region 1 (ITS1) was used to catalogue the species found in these communities. Functional profile of guilds found in the samples was associated to species using FUNGuild and variation in functional groups compared across sunlight exposure and altitude. Results revealed clear dominance of lichenized and stress-tolerant fungi in endolithic communities. The main variations in composition and abundance of functional groups among sites correlated to sun exposure, but not to altitude.

## 1. Introduction

Fungi play essential roles in the function of terrestrial ecosystems as contributors to carbon and nitrogen cycles. They can adopt a range of lifestyles, acting as saprotrophs, parasites, or symbionts (e.g., mycorrhizae, endophytes, and lichens), interacting with diverse breadth of organisms of all the biological kingdoms. Some fungal species are cosmopolitan, with a wide range of distribution. Other fungi have adapted to environments so harsh that most lifeforms are unable to grow. In extreme conditions where relatively few organisms are able to survive, fungi can play important role in the recycling of organic matter and enabling nutrient uptake.

Fungi, both filamentous and yeasts, dominate the eukaryotic composition of the highly oligotrophic soils of the Antarctic McMurdo Dry Valleys. The McMurdo Dry Valleys, the most similar environment available on Earth to the Martian surface, had been assumed to be practical sterile until a few decades ago, but has been found to support a limited number of living organisms [1,2,3,4]. The highest standing biomass of lifeforms in the Antarctic ice-free areas, including the McMurdo Dry Valleys, are found in rocky outcrops [5,6,7]. Rock in these areas is a predominant substrate for colonization, where the temperature, moisture availability, and UV exposure conditions approach the limits of tolerability, and the endolithic lifestyle is the best mode of survival for microorganisms inhabiting this extreme environment [8,9,10,11]. Endolithic communities in these Antarctica regions are populated by free living and lichenized fungi that form symbiotic associations with algae [9,12,13,14,15]. As border ecosystems, in constant, weak equilibrium between life and extinction, these communities are highly adapted but prone to external perturbations [14]; an increase of mean temperature, for instance, in the long run, may have irreversible effects, including extinction of endemic species [16]. Global warming is an unprecedented ecological, economic, and global health issue, and it is especially pronounced at high latitudes [17]. Antarctica, in particular, has experienced the most rapid changes in mean air temperatures on Earth over the past 50 years, reaching up to five times the mean rate of global warming in some areas [18]. It is predicted that these processes are likely to intensify in the future, and a deep understanding of Antarctic terrestrial ecosystems, both the diversity and functionality, is of utmost importance to developed tools and assays to monitor future changes [19]. From this perspective, the extremely adapted and highly sensitive endolithic communities of the Antarctic desert represent a perfect tool for both studying and monitoring the potential effect of global warming. In particular, we are focused on an investigation of the variation in biodiversity and functionality in these communities to test for biological variation in sensitivity to environmental pressure due to altitude and sun exposure. The models developed from these observations may be informative to get insights for predicting possible scenarios due to climate change [11,14]. Based on prior exploration of the pivotal role that fungi play in endolithic Antarctic communities, we tested how functional guild and biodiversity of species are found by altitude and sun exposure. The main functional groups of fungi in the Arctic can be structured at different spatial scales, from bioclimatic zones to habitat or even microhabitat scales, in response to environmental variability [20]. Results from these prior studies indicate that the composition of fungal communities is highly sensitive to variations in environmental conditions, but little is known about how the conditions control the role of fungal communities (i.e., their ecosystem function). Few studies that have analyzed functional groups of fungal communities along environmental gradients, and prior work has focused mainly in the Arctic [21,22,23,24], while little is known regarding Antarctic soils and rock communities.

In this study, we use a metabarcoding approach targeting the internal transcribed spacer region 1 (ITS1) to examine the distribution of fungal diversity across altitudinal gradient in Victoria Land, McMurdo Dry Valley, Antarctica. Rock samples collected from 834 to 3100 m a.s.l. with opposing sun exposures were analyzed to investigate how altitude and sun exposure have shaped community composition, taxon abundance, and distribution of functional groups of fungi in Antarctic endolithic communities.

## 2. Materials and Methods

### 2.1. Sampling Area

**Figure 1:**
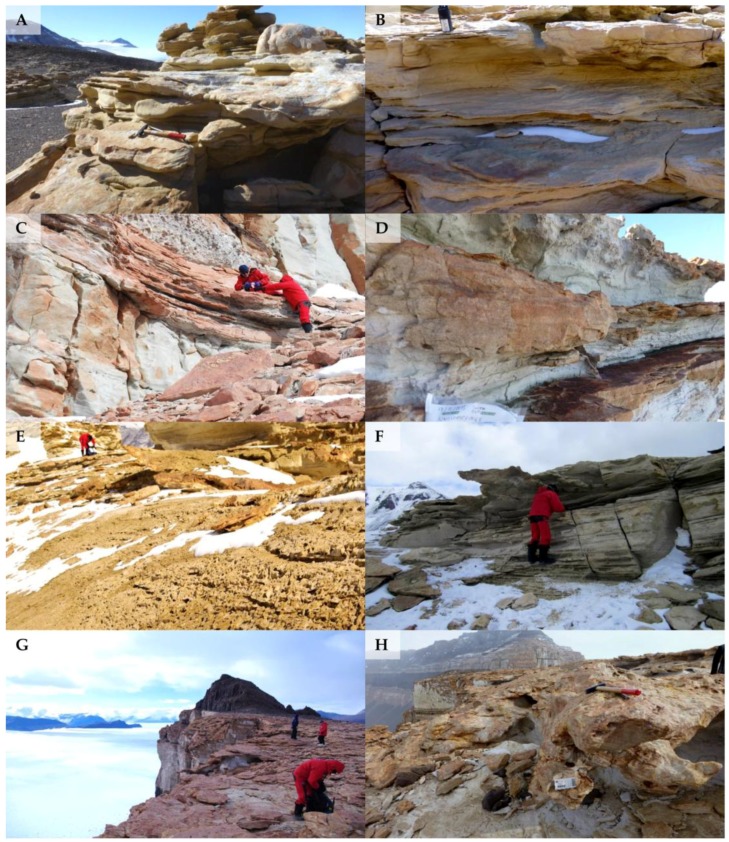
Localities visited in the McMurdo Dry Valleys (Southern Victoria Land), showing different sun exposition: (**A**,**B**) Battleship Promontory North and South, respectively; (**C**,**D**) University Valley North and South, respectively; (**E**,**F**) Siegfried Peak North and South, respectively; (**G**,**H**) Finger Mt. North and South, respectively.

Sandstone rock samples were collected in triplicate in Victoria Land (Continental Antarctica), along a latitudinal transect from 74°10′10.5′′ S 162°25′38.0′′ E (Timber Peak, Northern Victoria Land) to 77°54′43.6′′ S 161°34′39.3′′ E (Finger Mt., Southern Victoria Land) ranging from 834 m a.s.l. (Battleship Promontory, Southern Victoria Land) to 3100 m a.s.l. (Mt. New Zealand, Northern Victoria Land) (Table 1). In addition, rocks with different sun exposures were collected from four visited sites (Battleship Promontory, Siegfried Peak, Finger Mt. and University Valley) (Figure 1). All sites were visited during the XXXII Italian Antarctic Expedition (2015–2016). Rock samples were excised aseptically, transported, and stored at −20 °C at the Tuscia University (Viterbo, Italy) until processing.

### 2.2. DNA Extraction, Metabarcoding Sequencing, and Bioinformatic Analysis

Rocks were easily crushed using a Grinder MM 400 RETSCH (Verder Scientific, Bologna, Italy) in sterile conditions to avoid contamination. Metagenomic DNA was extracted from 0.3 g of rocks using MOBIO Power Soil DNA Extraction kit (MOBIO Laboratories, Carlsbad, CA, USA), according to the manufacturer’s instructions. ITS1F (CTTGGTCATTTAGAGGAAGTAA) [25] and ITS2 (GCTGCGTTCTTCATCGATGC) [26] primers were used to amplify the internal transcribed spacer 1 region (ITS1). PCR reactions were performed in a total volume of 25 μL, containing 1 μL of each primer, 12.5 μL of Taq DNA Polymerase (Thermo Fischer Scientific Inc., Waltham, MA, USA), 9.5 μL of nuclease-free water (Sigma-Aldrich, St. Louis, MO, USA) and 5 ng of DNA, following Coleine et al. [15]. PCR conditions were initial denaturation at 93 °C for 3 min, 35 cycles of denaturation at 95 °C for 45 s, annealing at 50 °C for 1 min, extension at 72°C for 90 s, followed by a final extension at 72 °C for 10 min in an automated thermal cycler (BioRad, Hercules, CA, USA). Amplicons, purified with Qiagen PCR CleanUp kit (Macherey-Nagel, Hoerdt, France) and quantified using the Qubit dsDNA HS Assay Kit (Life Technologies, Carlsbad, CA, USA), were tagged with unique barcodes to enable identification of each sample, and then pooled for run sequencing. Sequencing (paired-end reads, 2 × 300 bp) of the pooled libraries was performed on a single Illumina MiSeq flowcell at the Institute for Integrative Genome Biology, University of California, Riverside.

The ITS1 sequences datasets (dataset 1 includes samples from all localities along the altitudinal gradient; dataset 2 includes rocks from the four different exposed sites) were processed by AMPtk: Amplicon ToolKit for NGS data (formally UFITS) v.1.0.0 [27] according to Coleine et al. [15]. Briefly, the initial clean-up of the raw sequence data was carried out and the sequences were sorted according to samples barcodes after demultiplexing. Sequence regions of primers and adapters (identification tags) were removed from raw data. Reads were then subjected to quality trimming, PhiX screening, and removal of putatively chimeric sequences utilizing USEARCH with default parameters (v. 9.1.13) [28]. We opted for all global singletons (operational taxonomic units (OTUs) that were found only once across all samples) and rare taxa (<5 reads in all samples) were eliminated as likely false positives due to sequencing errors, as suggested by Lindahl et al. [29]. OTUs with less than 97% similarity to any identified fungal sequence were also excluded from the final analysis, as has been routinely applied in fungal ecology studies [30,31,32,33,34], and operational taxonomic units (OTUs) were identified using the VSEARCH (v 2.3.2) [35] algorithm. Finally, taxonomic identification was performed with hybrid database SINTAX/UTAX [28].

All primary amplicon sequence data is archived in NCBI SRA database linked to BioProject accession number PRJNA453198.

The ecological guild of the fungal OTUs was parsed using FUNGuild tools [36], which include a confidence ranking (“highly probable”, “probable”, and “possible”), reflecting the likelihood that a taxon belongs to a given guild. Since the calling of guilds in FUNGuild is predicated on confidence in the assigned taxonomy, 93% threshold has been chosen to represent a reasonable general cut-off point for ITS-based inputs. Assignments for functionality were based on assessments given in primary research literature and, where appropriate, alternative guild assignments where chosen, depending on own experience.

Venn diagrams of functional groups were constructed to show the number of shared OTUs among north and south sun exposition of the four selected localities (Battleship Promontory, University Valley, Siegfried Peak and Finger Mt.) using VENNY [37].

### 2.3. Statistical Analysis

For each functional group, richness in species (S), Shannon’s diversity index [38], and Simpson’s (1-D) dominance index [39] were calculated using Primer-E v7 software (PRIMER-E Ltd., Plymouth, UK) following Selbmann et al. [14].

Altitudes and biodiversity indices were compared by a two-way ANOVA to test for effects of altitude and pairwise multiple comparison procedure (Tukey test) carried out using the statistical software SigmaStat 2.0 (Jandel, Chandler Heights, AZ, USA) (*p* < 0.05). Statistics has been performed for each functional group. The non-parametric Spearman’s correlation coefficient was further calculated for each functional fungal group, and graphically represented to explore relationships between the biodiversity indices and sampled localities [40].

To avoid the uncertainties regarding the reliability of sequence reads abundance as indicator of taxon abundance or biomass in the samples [41,42], the effect of sun exposure was tested by evaluating changes in community composition of functional groups with non-metric multidimensional scaling (NMDS) based both on abundance data, calculating Bray–Curtis distance and presence–absence data, using Jaccard index and PAST v.2.17 software (PAleontological Statistics) [43]. Abundance data were square-root transformed to limit the influence of OTUs with high sequence counts. Analyses were carried out with 999 permutations.

Since changes in sequence counts can indicate relative changes in abundance [42], we have also compared per-OTU mean read counts across the north and south sun exposure groups to calculate mean effect size with 95% confidence interval for each functional group. A small probability *p*-value indicated a significant difference in diversity index between the two groups.

## 3. Results

### 3.1. Bionformatic Analysis and Guild Assignment

The multiplexed files contained 2,405,001 sequence reads for dataset 1 and 1,674,913 for dataset 2, resulting in 1,301,152 fungal ITS rRNA gene reads for dataset 1 and 978,632 for dataset 2, passing the quality trimming and filtering steps. After the singletons and rare taxa (<5 reads) were removed (123 out of 449 OTUs total in dataset 1 and 119 out of 270 OTUs total), sequences of dataset 1 were grouped into 326 operational taxonomic units (OTUs), while 151 OTUs were retrieved in dataset 2.

Ecological functions were assigned to 89 OTUs in dataset 1 and 110 OTUs in dataset 2. Based on taxonomic identities of the matching reference sequences, the functional classification was established as follows: (i) Lichenized (LIC), Lecanorales, Lecideales, and Acarosporales; (ii) Saprotrophs (SAP), which include mainly basidiomycetous yeasts species in the orders Tremellales and Sporidiobolales (genera *Cryptococcus*, *Naganisha*, and *Rhodotorula*) and ascomycetous yeasts in the genus *Taphrina*; (iii) rock-inhabiting fungi and black yeasts (RIF + BY), the first primarily consists of the Dothideomycetes (i.e., genera as *Friedmanniomyces*, *Extremus*, *Elasticomyces*, *Cryomyces*) and Eurotiomycetes, Chaetothyriales (*Knufia*), and secondarily Eurotiomycetes, Chaetothyriales (genera as *Exophiala*, *Capronia*, *Cladophialophora*); (iv) saprotrophs–plant pathogens (SAP + PP) were represented by few records (19 OTUs) of filamentous ascomycetes belonging to the genera *Cladosporium*, *Pseudogymnoascus*, *Fusarium*, *Leptosphaeria*, *Lachnum*. LIC class were the most numerous functional group, with 36 and 53 OTUs assigned in dataset 1 and 2, respectively, while SAP + PP had the fewest number of OTUs (10 and 9 in dataset 1 and 2, respectively) (Table 2).

### 3.2. Diversity Measures

Species richness (S), Shannon (H’), and Simpson (1-D) indices of each fungal functional group were computed and reported in Table 3. The LIC functional group had the highest average fungal richness (16 ± 4.7 OTUs), followed by RIF + BY (7.5 ± 2.9 OTUs), SAP (5 ± 1.7), while (SAP + PP) was comprised of only a few taxa (3.1 ± 0.9). The highest richness (25 OTUs) was observed at 1720 m a.s.l., while the lowest values were detected at 834, 2090, and 2702 m a.s.l. LIC represented the more biodiverse functional group, exhibiting Shannon’s index mean value 2.5 ± 0.31, and ranging from 1.95 (834 m a.s.l.) to 3.06 (1720 m a.s.l.); differently, SAP + PP group had the least diverse community based on the lowest H’ mean value (0.81 ± 0.39), with the minimum (0.23) at 1620 m a.s.l. LIC and RIF + BY fungi were the highest Simpson’s index values (mean 0.91 ± 0.02 and 0.82 ± 0.08, respectively) for richness.

**Figure 2:**
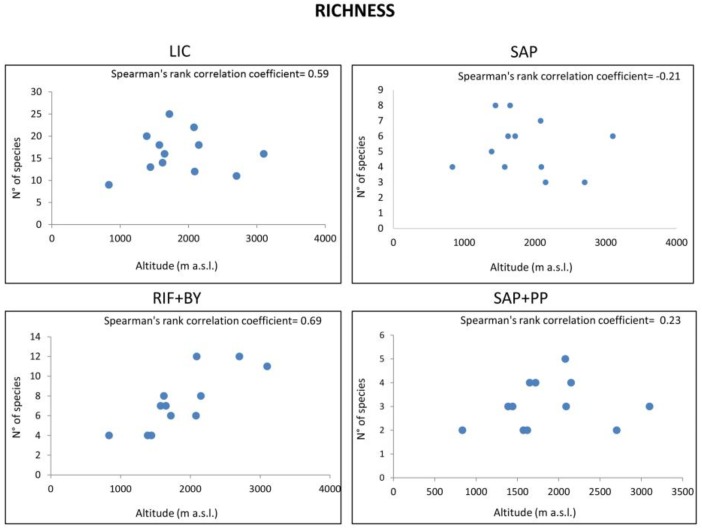
Spearman’s correlation coefficients between fungal richness of each functional group along an altitudinal gradient. *p >* 0.05 in LIC, SAP and SAP + PP panels; *p* < 0.05 in RIF + BY panel.

**Figure 3:**
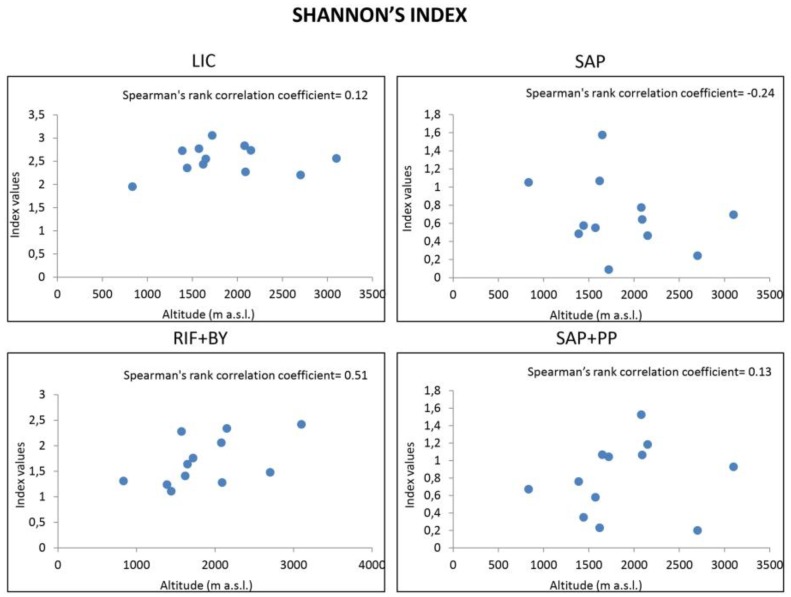
Spearman’s correlation coefficients between fungal biodiversity (Shannon’s index) of each functional group along an altitudinal gradient. *p >* 0.05 in all four panels.

Pairwise comparisons of calculated indices that were performed along the altitudinal gradient using Tukey’s test indicated that biodiversity varied significantly amongst sampled sites (*p* < 0.05, data not shown), even at similar altitudes, and also, amongst all functional groups within each community (*p* < 0.05, data not shown), highlighting that altitude had no global effect on fungal functional groups distribution. We also computed Spearman’s rank correlation coefficient (Figure 2 and Figure 3) to display eventual correlation between biodiversity indices and sampled sites; results showed that both S and H’ diversity indices were similar (*p >* 0.5) (Table 3), even though differences in altitude were significant (*p* < 0.05) (data not shown).

Altitude only affected richness in RIF + BY fungi (Spearman’s ρ value: 0.69, *p* < 0.05) (Figure 2); indeed, richness weakly increases with higher altitudes, even though Shannon’s index was unaffected (Figure 3).

### 3.3. Community Composition

To investigate the similarity of the fungal communities’ composition amongst different sun expositions, a non-metric multidimensional scaling (NMDS) ordination was computed both with the presence–absence matrix using the Jaccard dissimilarity index, and with the read-abundance data, using the Bray–Curtis index, to avoid the uncertainty whether read abundance was a good indicator of OTU abundance in the samples [41]. Since both approaches produced similar results, we showed results based on abundance only.

**Figure 4:**
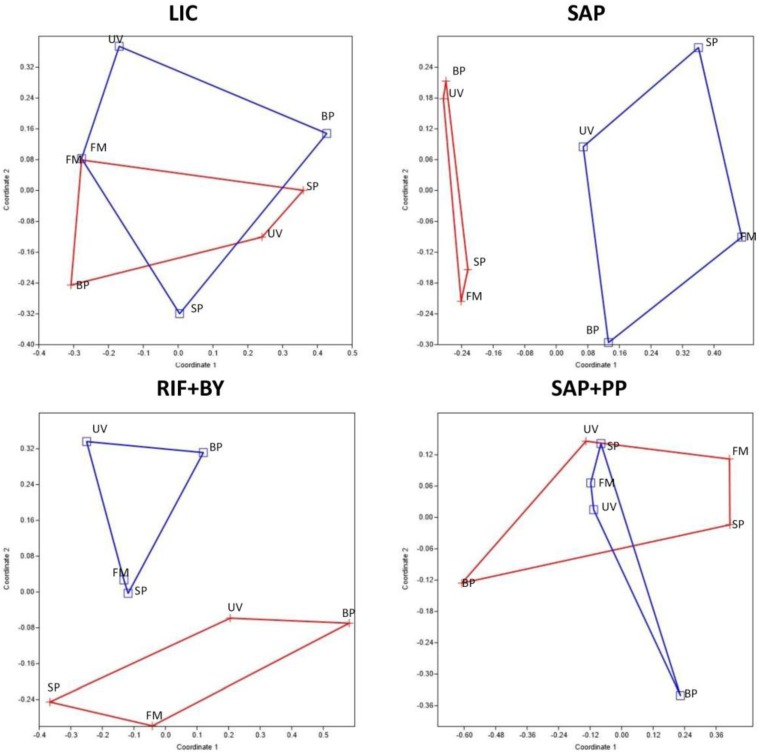
Non-metric multidimensional scaling (NMDS) ordination plots for each functional fungal group of Antarctic endolithic communities differently sun exposed (blue lines: south sun exposition; red lines: north sun exposition), based on square-root transformed abundance data.

NMDS analyses resulted in two-dimensional solutions with final stress values of 0.11 (LIC), 0.09 (SAP), 0.08 (RIF + BY), and 0.10 (SAP + PP), with final instability values less than 0.001. Globally, the NMDS plots revealed a strong structuring of fungal communities according to the sun exposition in the LIC, SAP, and RIF + BY functional groups (*p* < 0.05), while the sun exposure parameter did not significantly alter fungal community composition in the SAP + PP group (*p >* 0.05) (Figure 4).

Abbreviations are LIC = lichenized; SAP = saprotrophs; RIF + BY = rock-inhabiting fungi and black yeasts, and SAP + PP = saprotrophs–plant pathogens.

Localities are abbreviated as follows: BP: Battleship Promontory; SP: Siegfried Peak; FM: Finger Mt, and UV: University Valley.

Final stress values are: LIC: 0.11; SAP: 0.09; RIF + BY: 0.08; and SAP + PP: 0.10.

**Figure 5:**
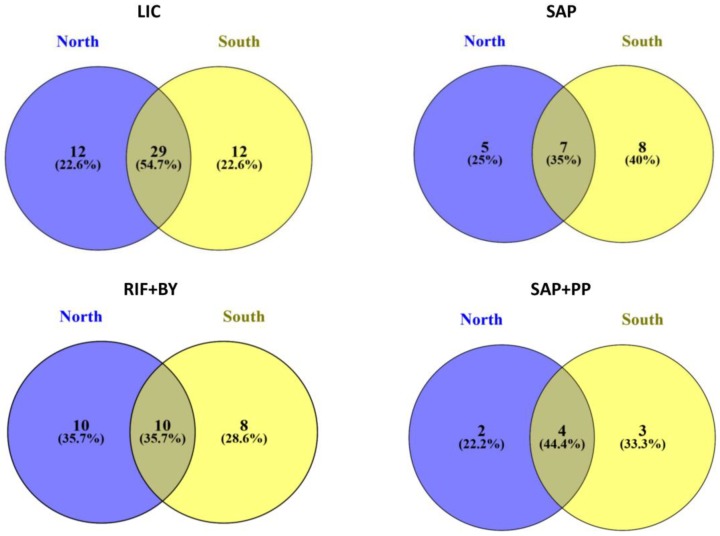
Venn diagram of the four functional groups of fungi showing the distribution of OTUs between north and south exposition. The abbreviations represent functional groups. Both the percentages of OTUs that were shared and found exclusively in each sun exposure are indicated.

Venn diagrams indicated that a substantial fraction of OTUs were unique to a sun exposition type: for example, more than half (54.7%) of the LIC-OTUs are shared among north and south exposure, while the 22.2% was unique; also, among the other three functional groups, a huge proportion of OTUs (ranging from 22.2 to 40%) was found exclusively in north or south sun exposition (in particular, 40% of SAP-OTUs were exclusive to south exposition only) (Figure 5).

The average sequence read count of OTUs among north and south exposition for each functional fungal group was different between the two exposures (data not shown). Trends of the mean sequence reads count per functional group indicated significant changes (*p* < 0.05), with a significant decrement in endolithic communities sampled in south exposition in LIC, SAP, and SAP + PP fungi (north exposition: LIC = 657,000, SAP = 856, and SAP + PP = 11,980; south exposition: LIC = 515,572, SAP = 523, and SAP + PP = 5410). Conversely, RIF + BY group showed a significant increase at south exposition, where 20,102 reads were observed, and 16198 were retained in north exposition.

## 4. Discussion

Since their discovery almost 40 years ago [8], the cryptoendolithic microbial communities of the Antarctic Desert have stimulated our imagination, giving the evidence that microbes may thrive and reproduce under conditions that were considered incompatible for life [1].

Despite recent advances, data on fungal biodiversity in endolithic communities remain still scant, primarily coming from investigations limited to single rock samples or from a single location [2,44,45]; only recently have broader surveys been undertaken [15]. The aim of this study was to test the organization, abundance, and variation in composition of functional groups of fungi in Antarctic endolithic communities. We sampled across a wide geographical region that spanned from Southern (Dry Valleys) to Northern Victoria Land, incorporating an altitudinal gradient (from 834 to 3100 m a.s.l.) and varied sun exposure, to gain insights on functional organization of community in response to these different selective and potentially stressful environmental conditions.

Although not all the OTUs in our datasets could be confidently assigned to species or genera, due to limited fungal reference sequences, we were able to assign the identified species to fungal functional groups based on the known ecology of reference taxa. Four groups of fungi were identified, taking into account the simple organization of these communities, relying on very short food chains constituted of primary producers and consumers/saprotrophs, and considering their relative fundamental ecological roles to maintain the equilibrium of the communities. Lichens are present inside rocks as the principal member responsible for carbon fixation, and can likely sustain the entire community. Based on this, we classified all fungi with known algal symbioses into the functional group of primary producers (indicated as LIC). Among four fungal functional groups, LIC represents the richest functional guild, and the most diverse group along the sampled locations. This high frequency for lichen group was not unexpected, since these obligate associations of fungi and algae are extremely successful worldwide, enabling these partnered organisms to spread over the harshest terrestrial environments, including the Antarctic desert [10]. The abundance of lichenized OTUs we observed is consistent with previous studies reporting lichens as extremely well adapted and widespread in Antarctica. The success of lichens is also thought to stem from their ability to be photosynthetically active at extreme temperatures [46]. Indeed, recent molecular surveys on lithic colonization patterns in Northern and Southern Victoria Land also revealed lichen mycobionts’ prevalence in rocks communities [14,15,46,47], with members belonging to the class Lecanoromycetes the only specimens recorded [48,49].

Basidiomycetous yeasts in the orders Tremellales and Sporoboridiales, and ascomycetous yeasts in the genus *Taphrina*, were rather abundant in this study, and have also been repeatedly isolated from these niches. Many species, such as *Cryptococcus friedmannii*, *C. vishniacii*, and *C. onofrii* (now reappraised in the genus *Naganisha*) [50], have been repeatedly reported in Antarctica, including cryptoendolithic communities, as well as associated to rocks in cold habitats worldwide [51,52,53,54,55]. The new species *Taphrina antarctica*, growing exclusively as a yeast, has been recently described from Antarctic cryptoendolithic communities. This genus has a biotrophic ecology as teleomorph, and it has been hypothesized that the fungus has focused on the saprotrophic part of its life cycle (anamorphic yeast) to successfully exploit the Antarctic rocky environment where plants are absent [56]. Due to their relative abundance and saprotrophic ecology in different niches, we found reasons that yeasts may play an important role as degraders in these communities too; for these reasons, we included all of them in the functional group of saprotrophs (SAP).

Rock-inhabiting fungi and black yeasts (RIF + BY) are also consumers but, because of their extreme resilience to stresses, including solar and UV irradiation, they are treated here as ecologically distinct from SAP. Rather, they are expected to play a primary role in the protection of the whole community forming a black barrier of “sunscreen” just above the photobiont stratification [57]. RIF + BY were recorded with high frequency and abundance, which was not surprising given that these organisms are among the most frequently isolated from Antarctic and Alpine lithic communities [12,13,58]. The RIF + BY encompass phylogenetically diverse fungi belonging to two main classes of the fungal kingdom Dothideomycetes and Eurotiomycetes. They show a notable stress-tolerance to chemical and physical injuries, such as extreme pH values, high and low temperatures, desiccation, UV and ionizing radiation, and even alpha particles [59,60,61,62,63,64,65]. Being specialized in the extremes [66], they normally occur in extreme habitats, from hot and cold deserts, rock surfaces and glaciers, saltpans, and acidic or polluted environments [8,61,67,68,69].

The last functional group includes filamentous saprotrophs and plant pathogens in the ascomycetes (SAP + PP). These fast growing ascomycetous fungi are among the most abundant guild detected in arctic soil communities [23,24] as, for instance, members of genus *Pseudogymnoascus* known to live as psychrotolerant saprotrophs [70]. Conversely, in this study, very few OTUs found from this guild (19 OTUs in all datasets) and have been isolated from Antarctic cryptoendolithic communities. For this reason, we treated them as separate from the other saprotrophs.

These communities display low biodiversity indices, confirming observations from recent molecular surveys of Antarctic lithic communities in Victoria Land [14,15]. Functional guilds showed variation in these indices with lichenized fungi having the highest (Shannon’s index ranging from 1.95 to 3.06), while the lowest diversity observed for the saprotrophic and saprotrophic–plant pathogenic fungal groups (Shannon’s index 0.83 to 1.84, and 0.23 to 1.52, respectively). These results indicated a high predominance of a restricted number species, and therefore, a notable degree of specialization. This conclusion is also supported by the Simpson’s index values (1-D), which is high, with a value of 0.90 (Table 3) for the LIC and RIF + BY functional groups. Antarctic endolithic communities are highly specialized ecosystems where few species are predominant [14]. This structure confers a consequently very low resilience to the community resulting in high vulnerability, so they may be dramatically affected by any external perturbations, even due to climate change [14,15].

The abundance and composition of functional groups of fungi did not present any patterns of correlation across locations and altitudes examined. The RIF + BY represented the only exception showing a weak positive correlation to altitude. Overall, the remarkable variability observed across altitudes indicates that it is not a primary factor shaping the composition and distribution of functional groups of fungi in the Antarctic endolithic communities.

Conversely, the overall effect of sun exposure was found to be significant, and most functional groups showed a clear trend in response to this variable. The primary effect encountered concerned OTU presence/absence in north and south sun exposed sites. Besides, differences recorded are not strictly a function of read count only, but also on composition and abundance, as suggested by NMDS ordination. In fact, relationship can be observed in the NMDS ordinations and Venn diagrams, even in the absences of differences in richness (Figure 4 and Figure 5). One expectation to this observation are saprotrophic–plant pathogenic fungi, which seem unaffected by this sun exposure. Changes in relative abundance of all functional groups between the two exposures were also evident. All groups were more abundant in communities sampled in north-exposed rocks, with the exception of RIF + BY, that invariably predominated in southern expositions where conditions are much more extreme. This finding is consistent with the peculiar ecology of these organisms that have high tolerance for low temperature and drought. In addition, they are weak competitors, due to their poor metabolic competences and slow growth, but are particularly successful when the spreading of others is hampered by the environmental constrains [57]. The increasing dominance of RIF + BY to the exclusion of other guilds of fungi in the face of environmental extremes makes them very suitable to define the border for life in these Mars-like environments, and to test the limits of habitability, a concept of astrobiological value [71].

This study represents a contribution to understanding complex relations between environmental parameters and functional fungal biodiversity in the endolithic communities of Antarctica. Future work should continue to test for the species functional group distribution pattern on an expanded sampling area, and explore additional locations in Victoria Land to confirm if the trends in species distributions in response to these environmental constrains is universal. Moreover, to get a complete picture of the functional responses of these communities, additional data collected on climatic and microclimatic trends, rock substratum features, and a fine-scale analysis on the responses of single taxon/species to stresses would need to be integrated. These data will enable tools better make predictions about the effects of climate change on these unique border ecosystems.

## Figures and Tables

**Table 1:** Table lists characteristics of 12 visited sites in Victoria Land: altitude, air temperature (measured when sampling), relative humidity, and geographic coordinates.

Site	Altitude (m a.s.l.)	Air Temperature (°C)	Humidity (%)	Coordinates
Battleship Promontory	834	−4.4	22.9	76°54′04.0′′ S 160°54′36.6′′ E
Trio Nunatak	1388	−5.1	40.9	75°28′56.6′′ S 159°35′28.3′′ E
Ricker Hills	1442	−7.2	42.7	75°42′14.6′′ S 159°13′39.4′′ E
Pudding Butte	1573	−8.5	32.4	75°51′30.2′′ S 159°58′25.7′′ E
Siegfried Peak	1620	−9.3	52.8	77°34′43.3′′ S 161°47′11.7′′ E
Linnaeus Terrace	1649	−9.6	58.6	77°36′01.3′′ S 161°05′00.5′′ E
Finger Mt.	1720	−6.4	35.1	77°54′43.6′′ S 161°34′39.3′′ E
Mt. Elektra	2080	−11.9	63	77°29′28.0′′ S 160°54′16.4′′ E
University Valley	2090	−14.3	18	77°52′28.6′′ S 160°44′22.6′′ E
Knobhead	2150	−12.5	50	77°54′37.8′′ S 161°34′48.8′′ E
Timber Peak	2702	−12.4	30.1	74°10′10.5′′ S 162°25′38.0′′ E
Mt New Zealand	3100	−17.2	47.6	74°10′44.0′′ S 162°30′53.0′′ E

**Table 2:** Number of OTUs retained for each fungal functional group in the two analyzed datasets.

N° of OTUs	Dataset 1	Dataset 2
LIC	36	53
SAP	19	20
RIF + BY	24	28
SAP + PP	10	9

LIC: lichenized; SAP: saprotrophs; RIF + BY: rock-inhabiting fungi and black yeasts; PP: plant pathogens.

**Table 3:** Diversity indices for fungal ITS rRNA gene sequencing were calculated on 12 endolithic communities. Species richness (S), Shannon’s index (H’) and Simpson’s index (1-D) values are reported for each functional group. Unidentified operational taxonomic units (OTUs) include those that could not be assigned to a guild.

Index	Altitude (m)	LIC	SAP	RIF + BY	SAP + PP	Unidentified OTUs
Richness (S)	834	9	4	4	2	13
	1388	20	5	4	3	15
	1442	13	8	4	3	11
	1573	18	4	7	2	12
	1620	14	6	8	3	15
	1649	16	8	7	4	13
	1720	25	6	6	4	14
	2080	22	7	6	5	15
	2090	12	4	12	3	11
	2150	18	3	8	4	9
	2702	11	3	12	2	11
	3100	16	6	11	3	8
Mean Value		16	5	7	3	12
Shannon’s Diversity (H’)	834	1.95	1.23	1.31	0.68	
	1388	2.78	1.2	1.24	0.76	
	1442	2.36	1.35	1.11	0.35	
	1573	2.77	0.98	2.28	0.58	
	1620	2.43	1.36	1.41	0.23	
	1649	2.55	1.84	1.64	1.07	
	1720	3.06	1.1	1.76	1.04	
	2080	2.84	1.31	2.06	1.52	
	2090	2.27	1.06	1.28	1.07	
	2150	2.73	0.85	2.34	1.19	
	2702	2.20	0.83	1.48	0.4	
	3100	2.56	1.38	2.42	0.93	
Mean Value		2.54	1.21	1.69	0.81	
Simpson’s Dominance (1-D)	834	0.86	0.98	0.83	0.57	
	1388	0.94	0.71	0.77	0.60	
	1442	0.91	0.74	0.73	0.58	
	1573	0.94	0.71	0.92	0.51	
	1620	0.91	0.79	0.72	0.58	
	1649	0.92	0.88	0.84	0.64	
	1720	0.96	0.57	0.80	0.65	
	2080	0.94	0.73	0.90	0.86	
	2090	0.90	0.71	0.70	0.71	
	2150	0.93	0.61	0.90	0.75	
	2702	0.89	0.57	0.85	0.75	
	3100	0.92	0.78	0.94	0.62	
mean value		0.92	0.73	0.86	0.65	

LIC: lichenized; SAP: saprotrophs; RIF + BY: rock-inhabiting fungi and black yeasts; PP: plant pathogens.
